# Spatiotemporal evolution of global population ageing from 1960 to 2017

**DOI:** 10.1186/s12889-019-6465-2

**Published:** 2019-01-30

**Authors:** Junming Li, Xiulan Han, Xiangxue Zhang, Sixian Wang

**Affiliations:** 1 School of Statistics, Shanxi University of Finance and Economics, 696 Wucheng Road, Taiyuan City, 030006 Shanxi Province China; 2 School of Earth Science and Resources, Chang’an University, Middle-section of Nan’er Huan Road, Xi’an City, 710064 Shaanxi Province China

**Keywords:** Spatiotemporal evolution, Global population ageing, Bayesian statistics

## Abstract

**Background:**

Population ageing is an increasingly severe global issue. And this has been posing challenges for public health policies and medical resource allocation There are various features of population ageing in different regions worldwide.

**Methods:**

All data were obtained from the health data of World Bank Open Data. Quantile linear regression was used to subtly measure the common variation tendency and strength of the global ageing rate and ageing population. The Bayesian space-time hierarchy model (BSTHM) was employed to assess the detailed spatial temporal evolution of ageing rate and ageing population in global 195 countries and regions.

**Results:**

Annual growth of the ageing (65 and above) rate occurred on six continents: Europe (0.1532%), Oceania (0.0873%), Asia (0.0834%), South America (0.0723%), North America (0.0673%) and Africa (0.0069%). The coefficient of variation of the global ageing rate increased from 0.54 in 1960 to 0.69 in 2017. The global ageing rate and ageing population increased over this period, correlating positively with their quantiles. Most countries (37/39) in Europe belong to the top level with regard to the ageing rate, including the countries with the greatest degree of ageing—Sweden, Germany, Austria, Belgium and the UK—whose spatial relative risks of ageing are 3.180 (3.113–3.214), 3.071 (3.018–3.122), 2.951 (2.903–3.001), 2.932 (2.880–2.984) and 2.917 (2.869–2.967), respectively. Worldwide, 44 low ageing areas which were distributed mainly in Africa (26 areas) and Asia (15 areas) experienced a decreasing trend of ageing rates. The local trends of ageing population in the 195 areas increased.

**Conclusions:**

The differentiation of global population ageing is becoming increasingly serious. Globally, all 195 areas showed an increasing local ageing trend in absolute terms, although there were 44 low-ageing areas that experienced a decreasing local trend of ageing rate. The statistical results may provide some baseline reference for developing public health policies in various countries or regions, especially in less-developed areas.

## Background

Population ageing is a global issue that is becoming increasingly severe. An increasingly ageing population is posing challenges for public health [1, 2]. A transformation of the major health threats has been occurring due to population ageing. In the early twentieth century, the major health threats were communicable diseases, infectious and parasitic diseases, which most often claimed the lives of infants and children. At present, non-communicable diseases—such as heart disease, arthritis and dementia—that usually affect adults and older people impose the greatest global health burden [1–3]. In other words, new disease patterns are forming as a result of population ageing. This can be described as part of an “epidemiologic transition” [4]. To cope with the challenges arising from population ageing, it is important to investigate both the static characteristics and dynamic features of population ageing.

The evolutionary process of population ageing in every country is significantly heterogeneous [5]. However, because population ageing is a global phenomenon [6], it is essential to study the spatiotemporal evolution of population ageing from a global perspective. The “epidemiologic transition” must lead to the changing composition of the disease burden [2] and, consequently, the adjustment of medical resource allocation and public health policies, especially health care [7]. In the context of globalization, every country or region should consider and respond to the issue of population ageing. Each country or region need self-position its relative static grade level of ageing in the world and comprehend total and local dynamic transformation of global ageing. This is the basis for developing health policies and acting to face the challenge of ageing. Some previous studies have investigated spatial differences or patterns of one or several countries. Rogers et al. studied the spatial differences of ageing and made a horizontal comparison between the United States, Italy, Japan and the UK [8], showing that the UK and the United States were in the most serious situation (final stages) in terms of population ageing, whereas Japan was in the initial stages, with Italy occupying a position somewhere in between. Shan et al. compared and analysed the characteristics of the spatial distributions of population ageing in China and Japan [9], concluding that differences in natural environment and economic development have a great impact on such differences. Wang et al. (2015) used China, the United States, South Korea and Japan as examples to classify and analyse the spatial distribution of ageing in different types of countries [10], also finding that economic differences lead to the polarisation of regional ageing. In addition to the spatial differences, population ageing also exhibits obvious temporal evolution. Sanderson and Scherbov analysed the evolution of the median age from 1800 to 1944 and predicted an ageing trend from 2000 to 2050 [11]. A report by the Population Division provided a description of global trends in population ageing and predicted that the proportion of older people, which was 8% in 1950 and 10% in 2000, would be 21% in 2050 [12]. Similarly, Wolfgang et al. projected changes in the level of ageing for the world population over the course of the twenty-first century [2].

Most previous studies have focused on population ageing from a spatial or temporal perspective, whereas few researchers have examined the evolution of global population ageing from a spatiotemporal perspective. Presently, only four studies explore spatiotemporal trends in population ageing. Marcela et al. analysed the space-time variability of the age structure of the European population from 1950 to 2010 [13]. Terry and Roberto [14] and Cecilia et al. [15] researched the recent spatiotemporal patterns of population ageing in Germany and Italy. Han and Li et al. explored the spatiotemporal evolution of Chinese population ageing from 1992 to 2015 [16]. To our knowledge, this research is the first to explore the recent spatiotemporal variation of worldwide population ageing from the point of view of both absolute and relative terms. The results generate overall spatial patterns and local trends of the ageing rate and population that can provide valuable references for relevant policy makers, public health managers and demographers and help to identify the focus of intervention implementations as well as optimal medical resource allocation.

## Methods

### Source data

The source data used in this paper include two datasets, the global ageing population and ageing rate, which refer to the total population aged 65 and above and the percentage of this group in the total population. The datasets were downloaded from the World Bank Open Data (https://data.worldbank.org/indicator), covering 195 countries and regions spatially and temporally from 1960 to 2017.

### Quantile regression model

A quantile regression model (QRM), which has a number of advantages compared to the ordinary least squares regression model, was employed to analyse the total changing trend of the ageing rate and population. QRM was first used as a ‘robust’ regression technique [17] that allows for estimation when the typical assumption of normality of the error term might not be satisfied. In such cases, it can be used to estimate the effect of the explanatory variable on the dependent variable at different quantile points [18]. Quantile regression estimates are more robust against outliers than ordinary least squares (OLS) regression. The distribution of the dependent variable must not be required to be normal in QRM. Moreover, QRM can deal with heteroscedasticity and allows a more comprehensive analysis of the relationships between variables. Accordingly, QRMs are widely used in statistics and econometrics. In our paper, its mathematical form can be expressed as follows:

1 $$ {\mathrm{y}}_{\mathrm{it}}={\beta}_0^{(p)}+{\beta}_1^{(p)}t+{\varepsilon}^{(p)} $$

where y_it_ represents the ageing rate or ageing population in the i-th country or region in t year; *p* is the quantile ranging from 0 to 1; ${\beta}_0^{(p)}$ and ${\beta}_1^{(p)}$ represent intercept and linear regression coefficients at the *p* quantile; and *ε*^(*p*)^ is the corresponding error term. Additionally, the estimation is performed as described by Koenker and Bassett [17] by minimising the following equation:

2 $$ \min \left(\sum \limits_{y_{it}\ge {\beta}_1^{(p)}t}p\left|{\mathrm{y}}_{\mathrm{it}}-{\beta}_1^{(p)}t\right|+\sum \limits_{y_{it}<{\beta}_1^{(p)}t}\left(1-p\right)\left|{\mathrm{y}}_{\mathrm{it}}-{\beta}_1^{(p)}t\right|\right) $$

The regression coefficient ${\beta}_1^{(p)}$ will differ depending on the specific quantile, *p*, being estimated. Hence, the corresponding variation tendencies of disparate quantile locations (containing median location) of the global ageing rate and population can be estimated by employing QRM. In this paper, the QRM estimation was implemented using the Statsmodels library of the Python programming language.

### Bayesian spatiotemporal hierarchical model

To explore the spatiotemporal trends of global population ageing from 1960 to 2017, a Bayesian spatiotemporal hierarchical model (BSTHM) [19] combining the Bayesian hierarchical model and a space-time interaction model [19, 20] was employed. In this paper, the global ageing problem is examined considering the relative amount and absolute quantity of the population simultaneously (i.e. ageing rate and ageing population). The unit of measure for the ageing population is set as ‘thousand persons’; hence, the observed variable, ageing population, can be regarded as a continuous variable in addition to the ageing rate. Consequently, normal distribution was employed as the likelihood distribution, expressed as follows:

3 $$ ageing\ rate:{y}_it\  Normal\left({?}_it{,?}^2\right)I\left(0,1\right). $$

4 $$ ageing\ population:{Y}_it\  Normal\left({\gamma}_{it},{\partial}^2\right)I\left(0,\right). $$

where y_it_ and Y_it_ represent the ageing rate and ageing population of the i-th country or region in the t-th year; *μ*_*it*_ and *γ*_*it*_ are the corresponding mean values; *σ*^2^ and *∂*^2^ are the corresponding variances; I(0, 1) denotes the range between 0 and 1; and I(0,) denotes the range of greater than 0. The corresponding spatiotemporal process model is:

5 $$ ageingrate:\mathit{\ln}\left({\mu}_{it}\right)={\alpha}^{(r)}+{s}_i^{(r)}+\left({\beta}_0^{(r)}t+{v}_t^{\left(\mathrm{r}\right)}\right)+{\beta}_{1i}^{(r)}t+{\varepsilon}_{it}^{(r)}. $$

6 $$ ageing\ population:\ln \left({\gamma}_{it}\right)={\alpha}^{(P)}+{s}_i^{(P)}+\left({\beta}_0^{(P)}t+{v}_t^{(P)}\right)+{\beta}_{1i}^{(P)}t+{\varepsilon}_{it}^{(P)}. $$

where *α*^(*r*)^ and *α*^(*P*)^, whose priors used non-informative prior distributions, are the basic fixed constants for global ageing rate and ageing population, respectively; ${s}_i^{(r)}$ and ${s}_i^{(P)}$ are the overall spatial relative risk of the ageing rate and ageing population globally; $\left({\beta}_0^{(r)}t+{v}_t^{\left(\mathrm{r}\right)}\right)$ and $\left({\beta}_0^{(P)}t+{v}_t^{(P)}\right)$ describe the general trend of the global ageing rate and ageing population from 1960 to 2017, containing a linear trend and a random effect; and ${\beta}_{1i}^{(r)}$ and ${\beta}_{1i}^{(P)}$ indicate the local ageing rate and ageing population trend in the i-th country or region from 1960 to 2017. The overall spatial relative risk and the local trend parameters, ${s}_i^{(r)}$, ${s}_i^{(P)}$, ${\beta}_{1i}^{(r)}$ and ${\beta}_{1i}^{(P)}$, whose prior distributions were assigned by the Besag York Mollie (BYM) model [21], represent a conditional autoregressive (CAR) normal prior form expressing the spatial structured and unstructured random effects. The spatial adjacency matrix adopts the first-order ‘Queen’ adjoining form. ${\varepsilon}_{it}^{(r)}$ and ${\varepsilon}_{it}^{(P)}$ represent the corresponding Gaussian random terms. The prior distributions of all random variables in the model are determined as a strictly positive half-Gaussian distribution [22], N_+∞_(0, 10).

In our study, Bayesian statistical estimation was implemented using WinBUGS [23]. Posterior distributions of all parameters in the model were acquired through Markov chain Monte Carlo (MCMC) simulations. The convergence of Bayesian statistics was assessed using a standard tool, the Gelman–Rubin statistical coefficient [24], calculated from two MCMC chains with different initial values; the closer the value is to 1.0, the better the convergence is. The Gelman–Rubin statistical value was less than 1.04 for all model parameters.

## Result

### Descriptive statistics

**Fig. 1:**
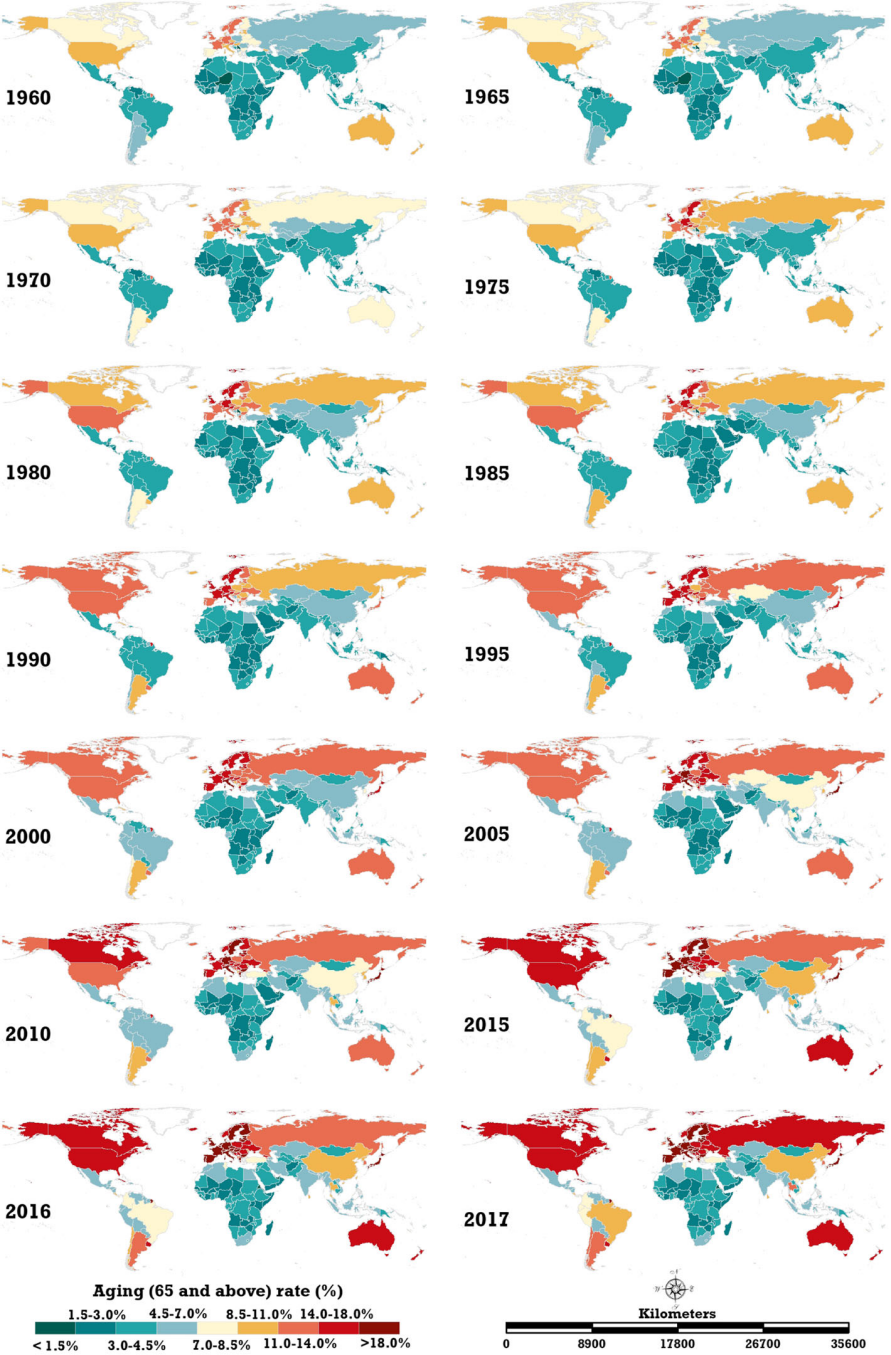
Spatial distribution of the global ageing rate from 1960 to 2017 (map generated with ArcGIS 10.3 by authors)

**Fig. 2:**
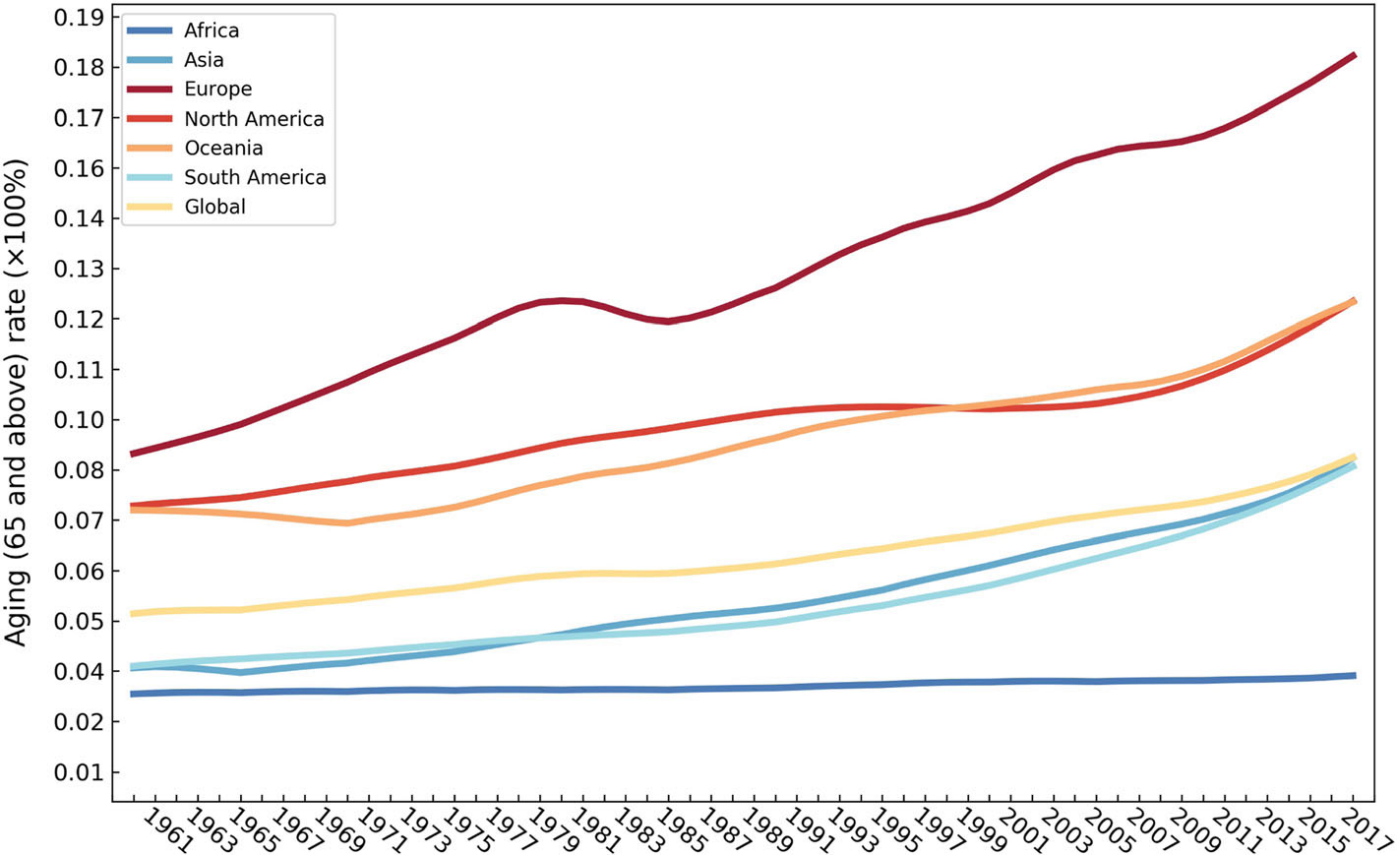
Global ageing rate trends for the continents of Africa, Asia, Europe, North America, South America and Oceania

The ageing rates in 195 countries and regions all demonstrated a growth trend. Figure 1 shows the spatial distribution of the global ageing rate from 1960 to 2017. The number of ageing countries and regions where the ageing rate is greater than 7.0% increased from 37 in 1960 to 91 in 2017. Moreover, the ranking of ageing countries changed during the study period. The top-five highest-ageing countries in 1960 were Austria (12.15%), Belgium (11.87%), United Kingdom (11.76%), Sweden (11.76%) and France (11.59%). In 2017, they were Japan (27.05%), Italy (23.02%), Portugal (21.50%), Germany (21.45%) and Finland (21.23%). Figure 2 illustrates the tendency of the ageing rates globally and on six continents. The ageing rates in Europe and Africa were always the highest and lowest among the six continents, respectively. Stronger increasing trends have been occurring in Europe (0.1532%), Oceania (0.0873%), Asia (0.0834%), South America (0.0723%) and North America (0.0673%) during this period, but not Africa (0.0069%). Moreover, the acceleration of the ageing rate on the five continents with higher ageing levels has increased since 2010. By 2017, the ageing rate of Europe, North America, Oceania, Asia, South America and Africa reached 18.26, 12.41, 12.40, 8.69, 8.48 and 3.49%, respectively. In the last 58 years, the ageing rates of Europe, North America and Oceania have been greater than the global ageing rate (8.70%), the ageing rate of Africa has been less than the global level and the ageing rates of Asia and South America have been nearly equal to the global level. Consequently, the six continents can be clustered into four classes: Europe, North America and Oceania, Asia and South America and Africa.

**Fig. 3:**
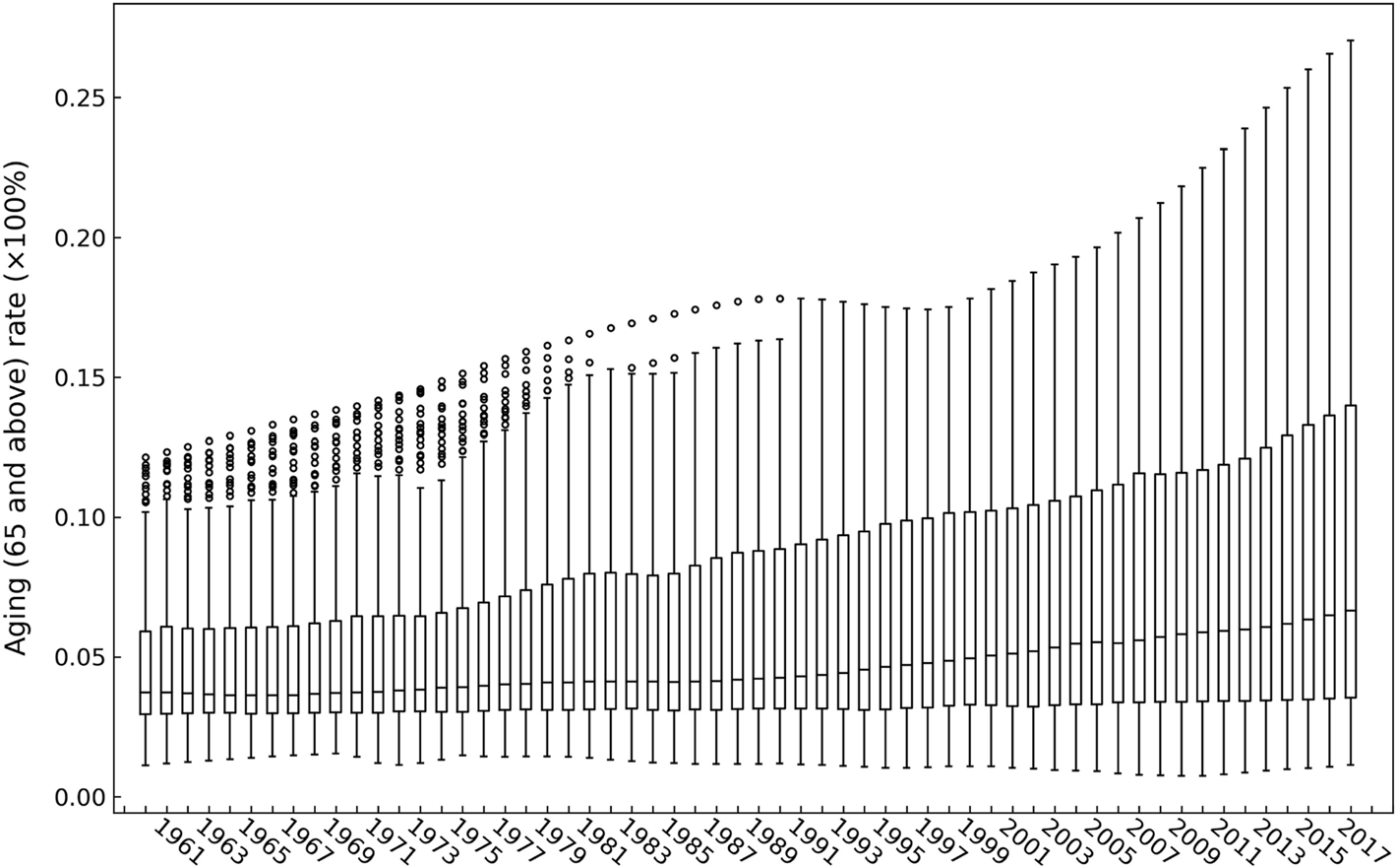
Boxplot of the global ageing rate for 195 countries and regions from 1960 to 2017

**Fig. 4:**
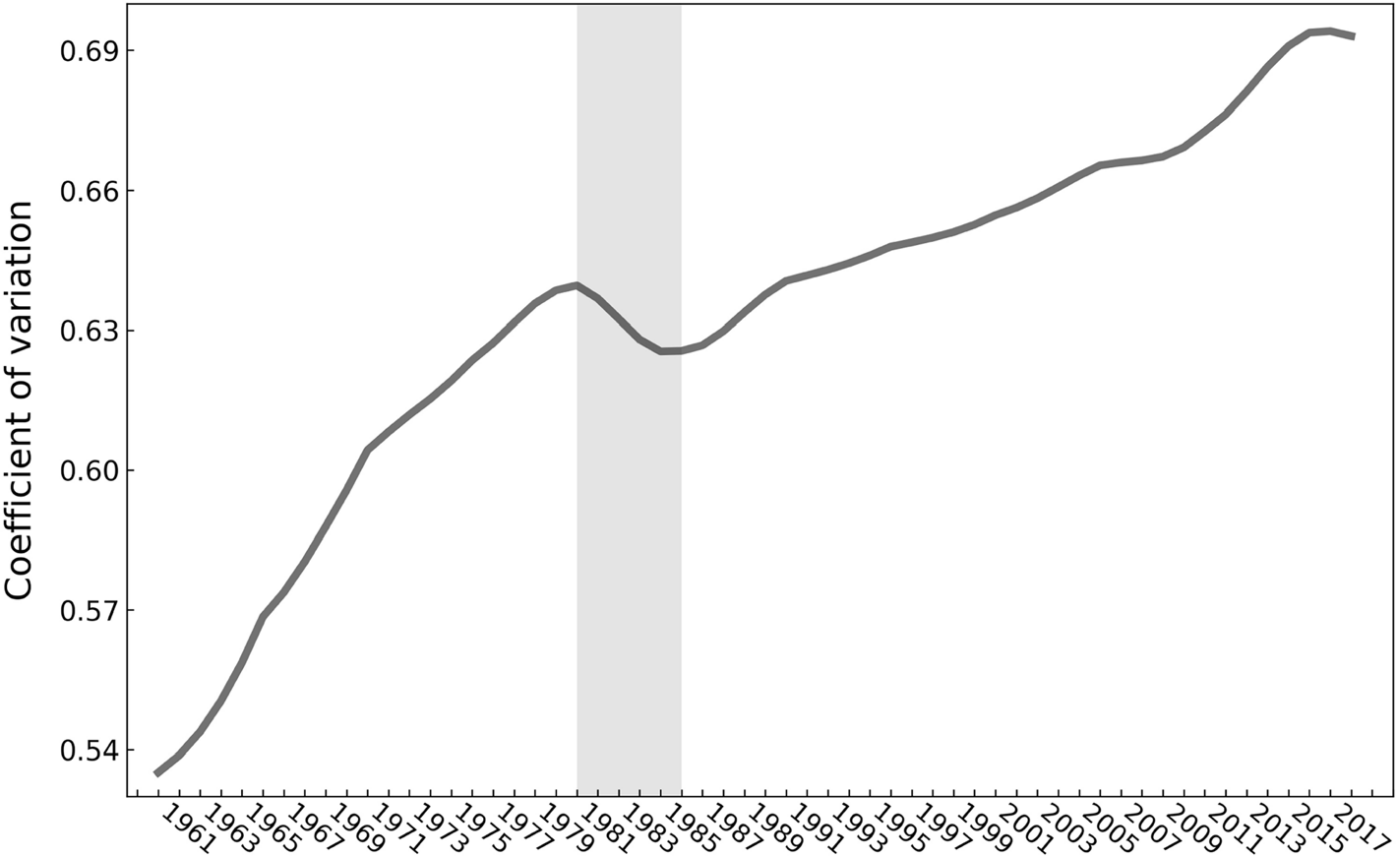
Trend of the coefficient of variation of the global ageing rate from 1960 to 2017

Figure 3 shows a boxplot of the global ageing rate from 1960 to 2017. The heterogeneity of the global ageing rate clearly increased, with the difference in ageing between various countries and regions continuously increasing during the 58-year period. Figure 4 shows the tendency of the coefficient of variation (CV) of the global ageing rate. The CV (calculated by dividing the standard deviation by the mean value) is the index for measuring the degree of heterogeneity in sample data. In statistics, heterogeneity is generally associated with a CV value greater than 0.15. The CV of the global ageing rate exhibited a strong increasing trend from 1960 to 1980. This was followed by a decreasing trend from 1980 to 1985 and then a subsequent increase from 1985 to 2016. The CV of the global ageing rate was 0.5352 in 1960 and grew to 0.6941 in 2016 and 0.6930 in 2017.

**Fig. 5:**
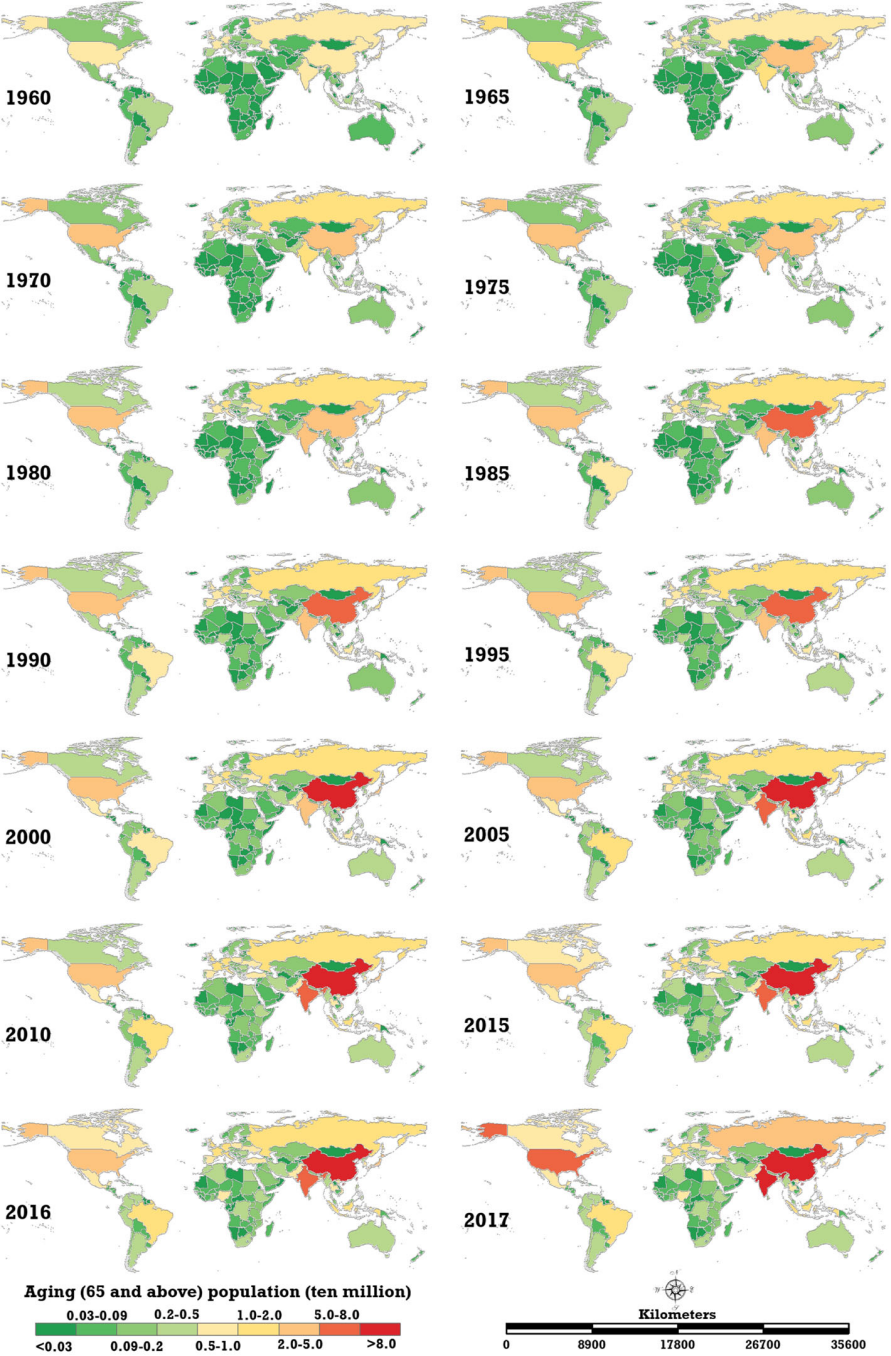
Spatial distribution of the global ageing population in 195 countries and regions from 1960 to 2017 (map generated with ArcGIS 10.3 by authors)

**Fig. 6:**
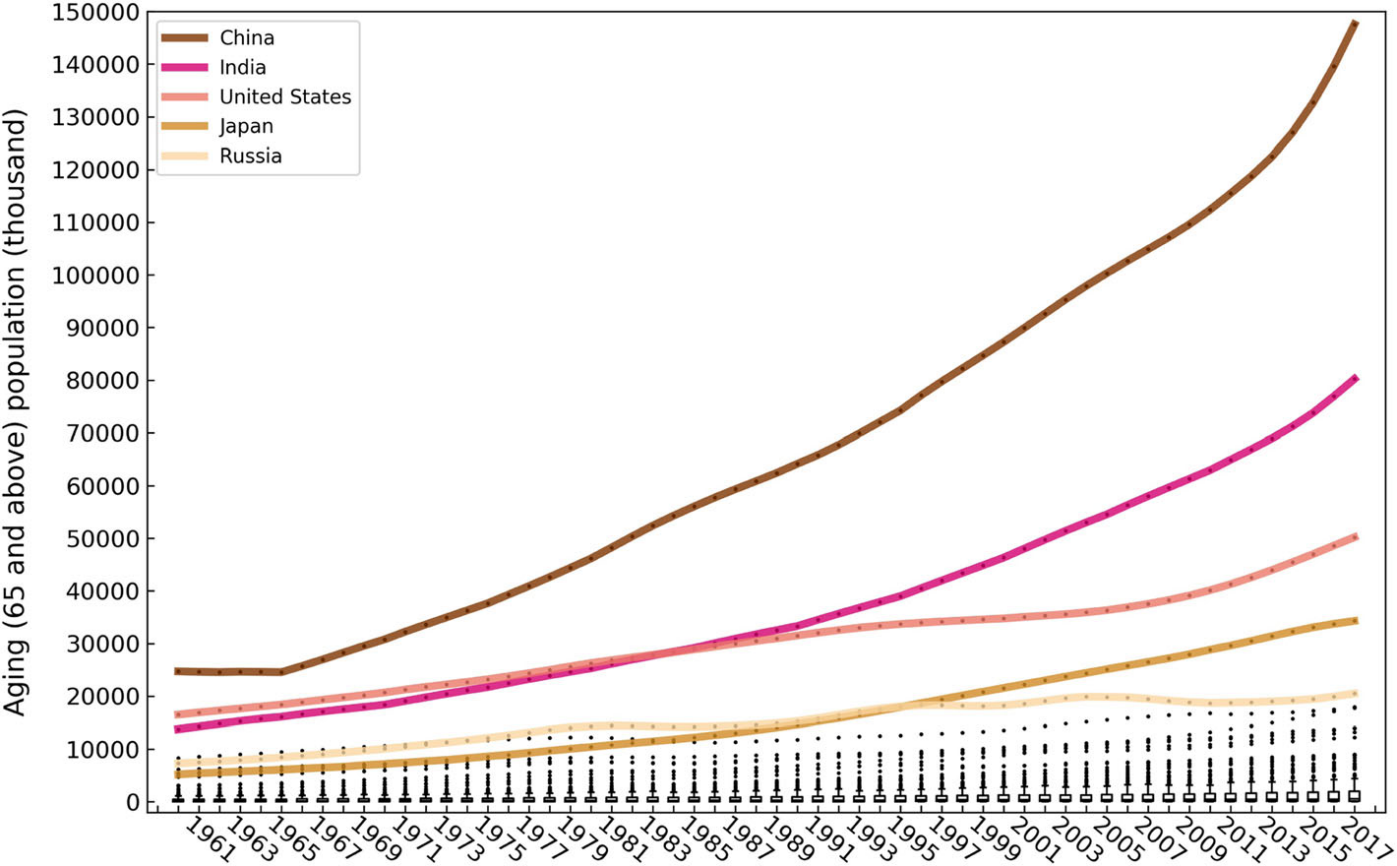
Boxplot of the global ageing population in 195 countries and regions from 1960 to 2017

In terms of absolute amount, the ageing population increased year by year in all 195 countries/regions, and the heterogeneity of the ageing population was greater than that of the ageing rate, increasingly consistently from 1960 to 2017 (from 3.42 in 1960 to 4.33 in 2017). Worldwide, the nations with large numbers of ageing populations usually also possessed a large total population, e.g., China, India, the United States, Japan and Russia. (Fig. 5). Figure 6 shows a boxplot of the global ageing population in this 58-year period, clearly illustrating this phenomenon. The differences in the five countries with the largest ageing populations amplified markedly during the study period. This was especially true for China, where the acceleration of the ageing population since 2014 was the fastest of any country in the entire study period, reaching 147.53 million in 2017 and accounting for 22.50% of the total global increase. The other four countries, India, the United States, Japan and Russia, had ageing populations of 80.20 million (12.23%), 50.20 million (7.66%), 34.29 million (5.23%) and 20.49 million (3.12%), respectively, in 2017. The global proportion of the ageing population of the top-five countries increased from 44.65% in 1960 to 50.74% in 2017. However, the countries with the largest ageing populations changed from 1960 to 2017: India and Japan jumped from the 3rd and 5th positions to become the nations with the 2nd and 4th largest ageing populations.

### Quantile regression result

**Fig. 7:**
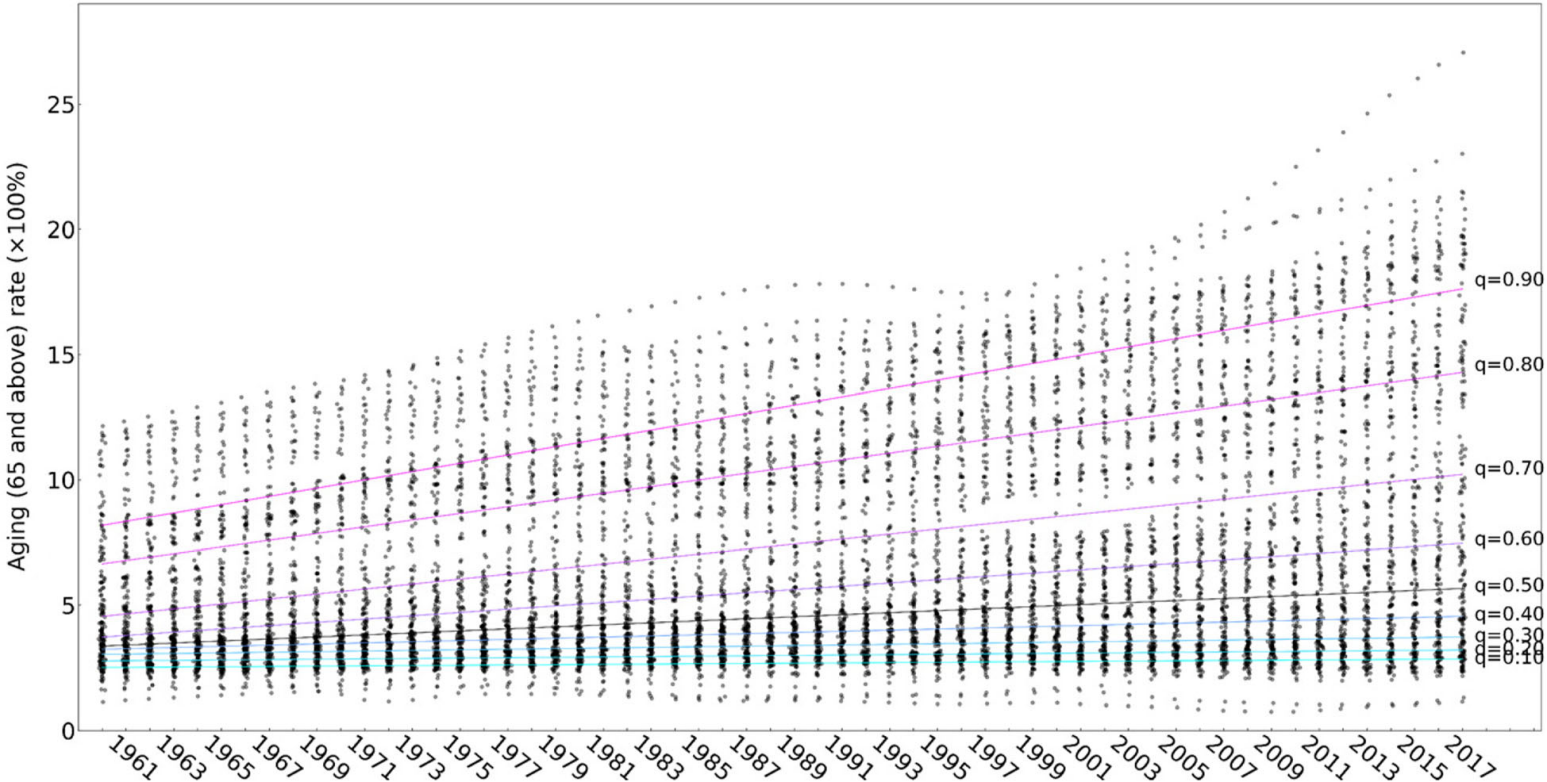
Quantile regression lines of the ageing rates in 195 countries and global regions from 1960 to 2017 with 9 quantiles—0.10, 0.20, 0.30, 0.40, 0.50 (median), 0.60, 0.70, 0.80 and 0.90—and the corresponding scatters

As the annual distribution of the global ageing rate and ageing population was not normal, our study employed QRM to quantificationally estimate the global linear growth of the ageing rate and ageing population. Figure 7 shows the quantile regression lines of the ageing rates in 195 countries and regions from 1960 to 2017 with 9 quantiles—0.10, 0.20, 0.30, 0.40, 0.50 (median), 0.60, 0.70, 0.80 and 0.90—and the corresponding scatters. The quantile regression coefficient correlates positively with the quantile (Table 1), Higher population ageing rates correspond to greater annual growth in population ageing rates. The median linear increasing global ageing rate is 0.040% (0.037, 0.044%). The annual increasing rates are up to 0.166% (0.157, 0.174%) and 0.134% (0.124, 0.144%) at the 90% quantile. However, the ageing rates increase 0.006 and 0.008 percentage points at the 10 and 20% quantiles.

**Table 1:** Quantile linear regression of the coefficients of the ageing rates in 195 countries and global regions from 1960 to 2017 with 9 quantiles: 0.10, 0.20, 0.30, 0.40, 0.50 (median), 0.60, 0.70, 0.80 and 0.90

Quantile	Intercept	Quantile linear regression coefficient (95% CI)	*P*-value
0.10	2.498	0.006 (0.004,0.007)	0.000
0.20	2.759	0.008 (0.006,0.009)	0.000
0.30	3.017	0.012 (0.010,0.014)	0.000
0.40	3.212	0.023 (0.020,0.026)	0.000
0.50	3.322	0.040 (0.037,0.044)	0.000
0.60	3.655	0.066 (0.060,0.071)	0.000
0.70	4.449	0.099 (0.087,0.112)	0.000
0.80	6.514	0.134 (0.124,0.144)	0.000
0.90	8.010	0.166 (0.157,0.174)	0.000

**Fig. 8:**
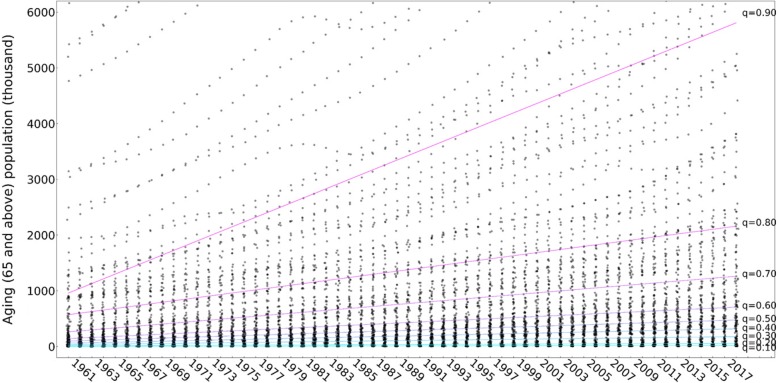
Quantile regression lines of the ageing population rates in 195 countries and global regions from 1960 to 2017 with 9 quantiles—0.10, 0.20, 0.30, 0.40, 0.50 (median), 0.60, 0.70, 0.80 and 0.90—and the corresponding scatters

The quantile regression coefficients of the global ageing population also increase as the quantile increases (Table 2). The difference in the ageing population is greater than that of the ageing rate across the world. This can be seen by comparing Figs. 3 and 6. Figure 8 and Table 2 show, respectively, an illustration and regression coefficients of the quantile linear regressions of the global ageing populations in 195 countries and regions from 1960 to 2017 with 9 quantiles. The regression coefficient (85.072 thousand persons per year) in the 0.90 quantile is 12.833 times greater than the median regression coefficient (6.629 thousand persons per year). It should be noted that the four countries with the largest ageing populations—China, India, the United States and Japan—experienced a more rapid increase in the rate of ageing than the other countries and regions. In the most recent five-year period (2013–2017), faster increases occurred in the three most populous countries (China: 6275 thousand persons per year; India: 2820 thousand persons per year; the United States: 1563 thousand persons per year) than in the earlier period (1960–2013). Japan has maintained a more rapid growth rate of 743 thousand persons per year since 1992. Although Russia is the country with the fourth largest ageing population since the beginning of the twenty-first century, the trend in the country has not increased; that is, the ageing rate remained stable.

**Table 2:** Quantile linear regression coefficients of the ageing population rates in 195 countries and global regions from 1960 to 2017 with 9 quantiles: 0.10, 0.20, 0.30, 0.40, 0.50 (median), 0.60, 0.70, 0.80 and 0.90

Quantile	Intercept	Quantile linear regression coefficient (95% CI) (thousand persons per year)	P- value
0.10	1.859	0.307 (0.139, 0.476)	0.000
0.20	13.430	0.732 (0.510, 0.954)	0.000
0.30	32.328	2.501(2.207, 2.795)	0.000
0.40	54.855	4.541(4.165, 4.918)	0.000
0.50	91.531	6.629 (6.121, 7.136)	0.000
0.60	132.930	9.902 (9.083, 10.721)	0.000
0.70	245.660	17.506 (15.654, 19.359)	0.000
0.80	542.945	27.862 (25.067, 30.656)	0.000
0.90	874.043	85.072 (76.170, 93.975)	0.000

### Bayesian statistical estimates

#### Overall spatial trend

**Fig. 9:**
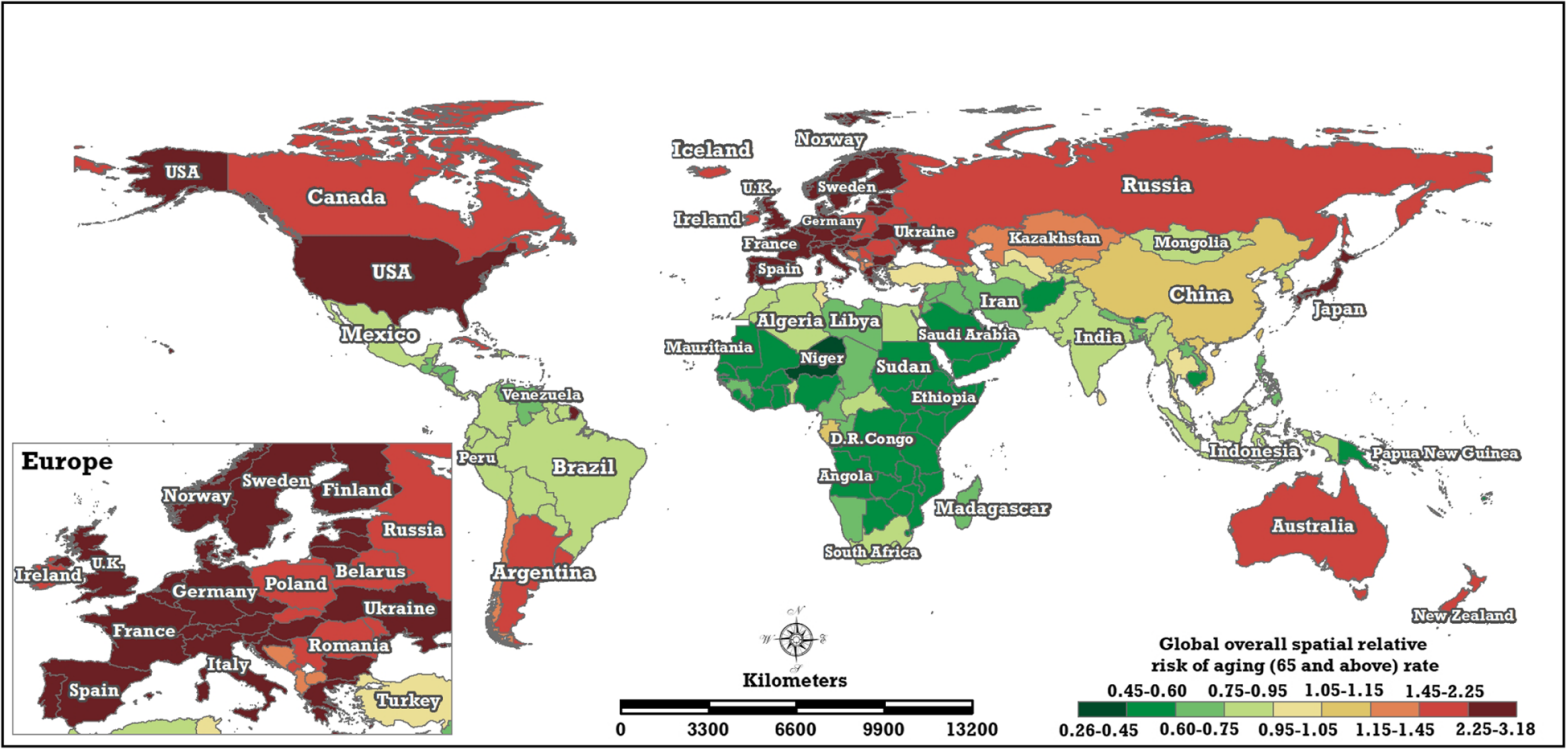
The overall spatial relative risks of the global ageing rate (the posterior median estimate of $\exp \left({\mathrm{s}}_{\mathrm{i}}^{\left(\mathrm{r}\right)}\right)$ of the BSTHM) (map generated with ArcGIS 10.3 by authors)

The overall spatial trend of the global ageing rate (Fig. 9) shows a steady spatial pattern, considering the space-time variation process from 1960 to 2017 synthetically, measured quantitatively with common spatial relative risk, i.e., the coefficient $\exp \left({s}_i^{(r)}\right)$, whose value indicates the magnitude of the ageing rate of the i-th country or region relative to the global average ageing rate, exp(*α*^(*r*)^). If $\exp \left({s}_i^{(r)}\right)>1.0$, the ageing rate of the i-th country or region is $\exp \left({s}_i^{(r)}\right)$ times the global common level, and vice versa.

The 195 countries and regions may generally be divided into five grades according to Jenks natural breaks classification [25] of the ageing rate level, namely, the posterior median estimate of $\exp \left({s}_i^{(r)}\right)$ from BSTHM: lower (0.26–0.60), low (0.60–0.95), middle (0.95–1.05), high (1.05–1.45) and higher (1.45–3.18). There are 55 countries and regions with higher ageing levels, 37 of which are located in Europe, including the top five countries, Sweden (3.180, 95% highest posterior density (95% HPD): 3.113–3.214), Germany (3.071, 95% HPD: 3.018–3.122), Austria (2.951, 95% HPD: 2.903–3.001), Belgium (2.932, 95% HPD: 2.880–2.984) and the United Kingdom (2.917, 95% HPD: 2.869–2.967). Eight countries are located in Asia, including Japan (2.291, 95% HPD: 2.235–2.347) and Georgia (2.015, 95% HPD: 1.966–2.066). Six are located in North America, including the United States (2.281, 95% HPD: 2.233–2.331) and Canada (2.079, 95% HPD: 2.026–2.134) etc., while Uruguay (2.189, 95% HPD: 2.136–2.240) and Argentina (1.671, 95% HPD: 1.623–1.722) are located in South America. The other two countries, Australia (2.090, 95% HPD: 2.041–2.142) and New Zealand (2.058, 95% HPD: 2.012–2.110), are located in Oceania. Twenty-four countries and regions are mainly distributed in Asia (11, including China (1.081, 95% HPD: 1.028–1.133), Korea (1.059, 95% HPD: 1.007–1.118), Singapore (1.060, 95% HPD: 1.004–1.112)) and North America (8), all with high ageing levels. Most countries and regions in Africa and some countries and regions in Asia and South America (including India and Indonesia) have low or lower ageing rates (green or dark green areas in Fig. 9).

**Fig. 10:**
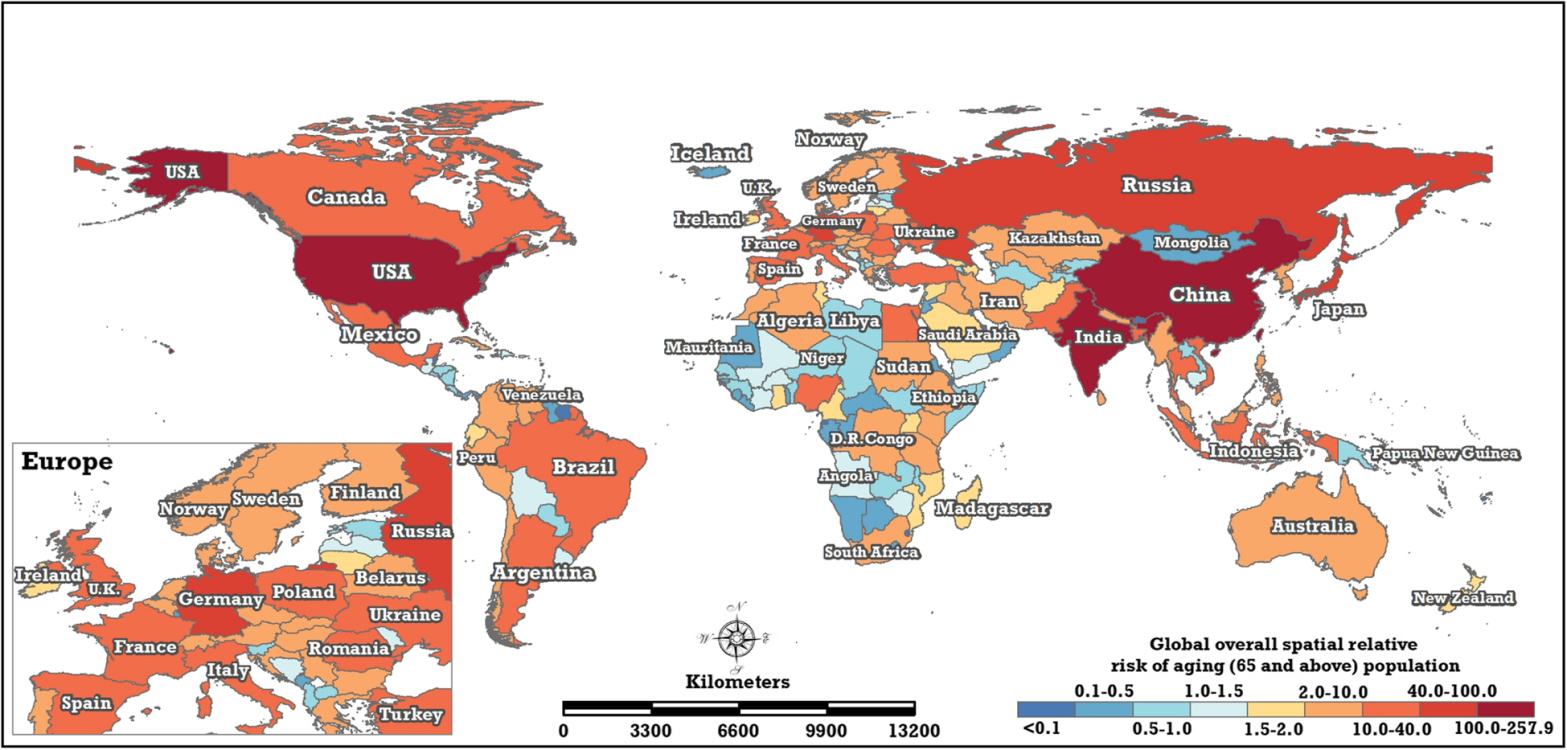
The common spatial relative risks of the global ageing population (the posterior median estimate of $\exp \left({\mathrm{s}}_{\mathrm{i}}^{\left(\mathrm{p}\right)}\right)$ of the BSTHM) (map generated with ArcGIS 10.3 by authors)

In this paper, the common spatial relative risk of the global ageing population (Fig. 10) was estimated using BSTHM. As shown in Fig. 9, the United States has both the highest ageing level ($\exp \left({s}_i^{(r)}\right)=2.28;$15.41% in 2017) and the largest ageing population ($\exp \left({s}_i^{(p)}\right)=126.40;$50,204,174 ageing persons in 2017) in the world. Nine countries in Europe (e.g. Sweden, Germany, Austria, the United Kingdom, Italy) have both high ageing levels ($\exp \left({s}_i^{(r)}\right)>2.01$) and large ageing populations ($\exp \left({s}_i^{(p)}\right)>10.31$). Additionally, another four countries, Canada, Japan, Russia and Argentina, have similar characteristics. However, there are eight countries (India, Indonesia, Bangladesh, Pakistan, Brazil, Mexico, Nigeria and Egypt) that have both large ageing populations ($\exp \left({s}_i^{(p)}\right)\ge 10.62$) and lower ageing levels ($\exp \left({s}_i^{(r)}\right)\le 0.55$). This is most obvious for India, which had an ageing population of 80,203,086 persons and an ageing rate and 5.99% in 2017; the corresponding $\exp \left({s}_i^{(r)}\right)$ and $\exp \left({s}_i^{(p)}\right)$ are 0.78 and 141.10.

#### Local trends

**Fig. 11:**
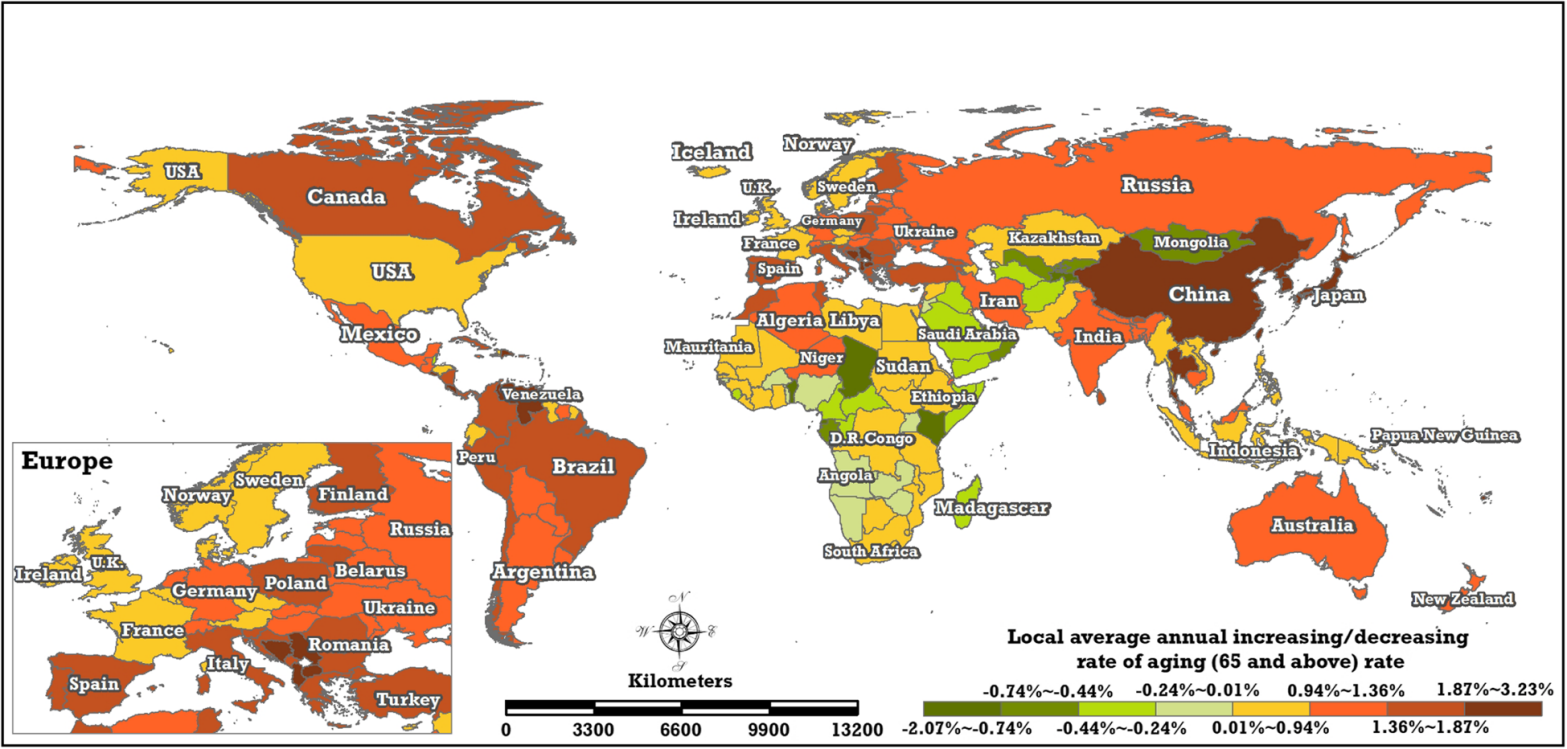
Local average annually increasing 496 or decreasing rate of ageing rate (the posterior median of $\exp \left({\beta}_0^{(r)}+{\beta}_{1i}^{(r)}\right)$ in the BSTHM) in the 195 countries and regions from 1960 to 2017 (map generated with ArcGIS 10.3 by authors)

**Fig. 12:**
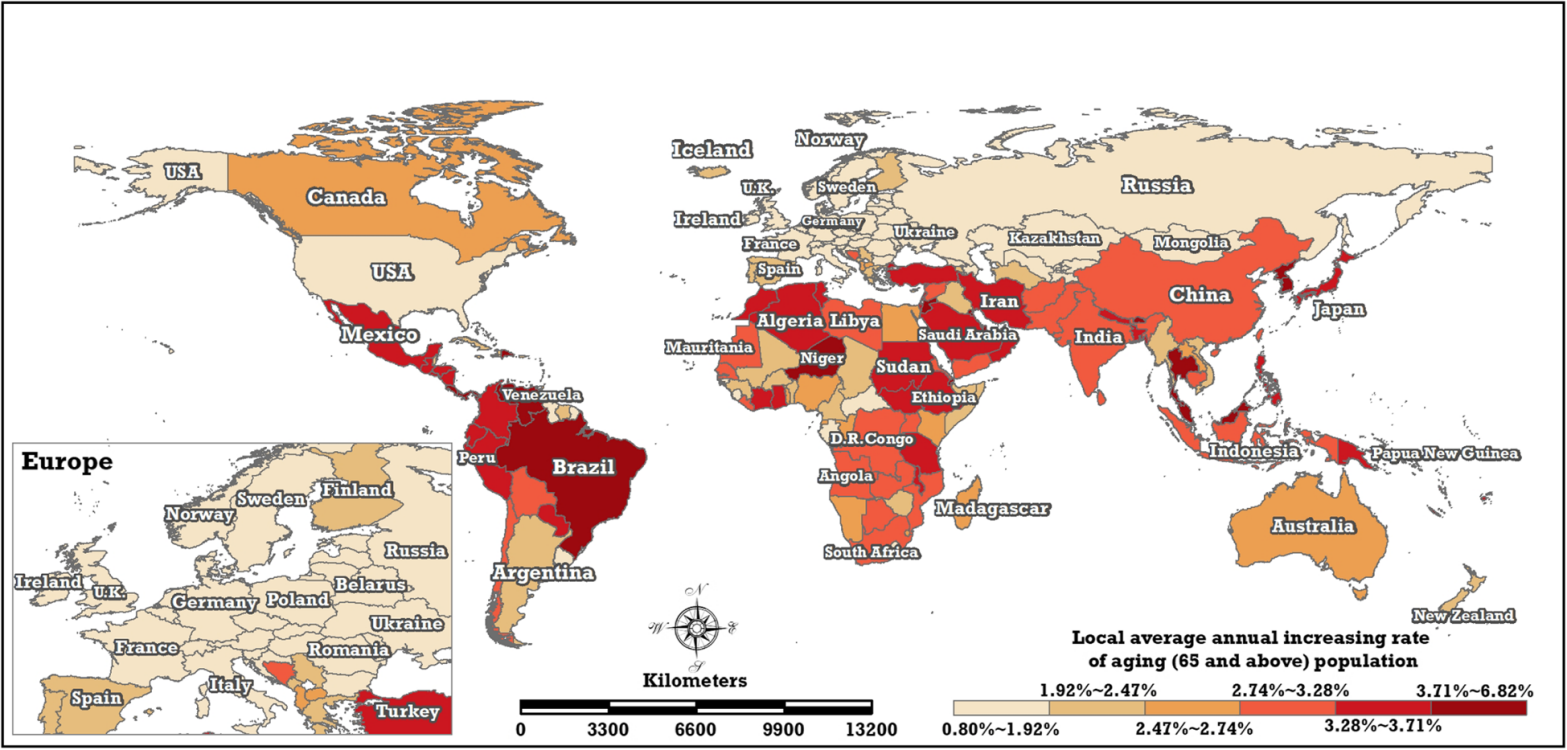
Local average annually increasing ageing population (the posterior median of $\exp \left({\beta}_0^{(p)}+{\beta}_{1i}^{(p)}\right)$ in the BSTHM) in the 195 countries and regions from 1960 to 2017 (map 501 generated with ArcGIS 10.3 by authors).

The local trends quantitatively measure the magnitude of change in every country or region based on spatially structured and unstructured effects. The average annual variational rate of the ageing rate and ageing population in each country or region from 1960 to 2017 can be estimated from the parameters, exp.(${\beta}_0^{(r)}+{\beta}_{1i}^{(r)}$) and exp.(${\beta}_0^{(p)}+{\beta}_{1i}^{(p)}$), of the BSTHM. Figures 11 and 12 show the distribution of the local average annual increasing or decreasing rates of the ageing rate and ageing populations in 195 countries and regions from 1960 to 2017, respectively.

Worldwide, 44 countries and regions (green-coloured areas in Fig. 11), which are distributed mainly in Africa (26 areas) and Asia (15 areas), have been experiencing a decreasing trend of ageing and low ageing levels, although they have a weakly increasing trend in their ageing populations (Fig. 12). The 71 other low or lower levelled countries and regions (e.g. Algeria, Niger, Morocco, Tunisia, Venezuela, Brazil, Peru, Colombia, India, Iran and Indonesia), whose spatial relative risk of ageing rate is less than 0.95, located mainly in Africa, South America and Asia, have been experiencing positive average annual growth in ageing rates.

Nine countries in Europe (France, the United Kingdom, Norway, Sweden, Austria, Ireland, Denmark, Belgium, Czech Republic) as well as the United States and Japan have the highest ageing rates in the world, but the local trends in these countries differ. These nine European countries and the United States have smaller average annual ageing growth rates (0.01%~ 0.94%) than Japan, where the annual increase in the ageing rate is very high. Other countries in Europe (e.g. Germany, Spain, Finland, Poland, Italy, Russia) exhibit a stronger increasing trend (0.94%~ 2.97%) in their ageing rates. While China is the most populous country in the world, its ageing level does not differ from that of other countries, on average. Additionally, a larger local increasing trend can be seen in three countries with high ageing rates: Canada, Australia and New Zealand.

Between 1960 and 2017, the estimated average annual growth rates of the ageing populations of all the studied countries and regions were positive. In terms of the local trends in the ageing populations, an obvious spatial pattern with a north–south partition (‘low in north, high in south’) can be seen. The local trends in Europe and North America were low annual increases in the ageing populations. The absolute annual increase in the quantity of ageing persons in China and India was larger than in all other areas, but the average annual increase rate was only medium high (2.74%~ 3.28%) relative to the rest of the world.

## Discussion

Population ageing is an unprecedented and pervasive phenomenon in nearly all countries and regions, regardless of whether they are developed or developing. Globally, falling fertility rates and significant improvements in life expectancy result in continuous population ageing. The remarkable increase of the proportion of the aged population is part of a shift in the leading causes of disease and death [1]. Currently, longevity has become the major risk factor for chronic killer diseases, such as cancer, cardiovascular diseases and neurodegenerative diseases [26]. Consequently, the composition of the global burden of diseases has been altered [2]. On the brink of this demographic milestone, public health policies and health systems will also need to be transformed. Fortunately, the aging problem has drawn considerable attention from international communities. The World Assembly on Aging was held in Vienna in 1982 and again in Madrid in 2002. Still, according to the World Health Organization (WHO) Study on global AGEing and adult health (SAGE), most countries have been slow to generate and use evidence to develop an effective health response to population ageing and the “epidemiologic transition.”

The global patterns of the overall spatial relative risk in the ageing rate exhibit a distinct geographical heterogeneity coinciding with the spatial pattern of development in 195 countries and regions. To test the association between the overall spatial relative risk of ageing rate and economic development in 195 countries, the geographical detector statistics [27, 28] was conducted. The statistical result showed that the q-value between the overall spatial relative risk of ageing rate and GDP per capita was 0.5036 (*P* < 0.001). It indicates that the level of population ageing in a nation is associated tightly with its economic conditions. Developed nations located majorly in the northern mid-latitudes, e.g., the United States, Canada and European countries, have the highest population ageing levels. Less-developed countries, e.g., African countries and western Asian countries, have lower population ageing levels. Developing countries, including China, India, Mexico and Brazil, have medium levels of population ageing. Population ageing arises mainly from decreasing fertility and increasing life expectancy [6]. In general, better economic conditions provide better health systems and medical resources, which are the preconditions of increasing life expectancy. The overall spatial relative risk in the ageing rate estimated by BSTHM revealed this point. Some previous studies research the population ageing of one or some nations. Jagger et al. [29] reported that European countries have the longest life expectancy in the world, and Kinsella et al. [5] found that the speed of population ageing of some European countries, France, Sweden and the UK, is slower than that of some East Asian countries, south Korea, China and Thailand. Marcela et al. [13] stated that the present ageing level of Europe is the most pronounced among the continents. These findings coincide with our findings. In 2001, Eberstadt reported that America’s population was younger than that of other developed countries, because of higher fertility rates and immigration [30]. However, based on the population data from 1960 to 2017, this study found that the overall spatial relative risk and local trends of the population ageing rate of the United States was the same as in European countries. The problems of the population ageing in the three populous Asian countries—China, India and Japan—were also studied. China and India have experienced rapidly decreasing fertility since the 1970s, and the life expectancy in China has increased significantly as a result of rapid economic growth and the development of public health and medical systems [31–33]. Hence, China and India are expected to experience fast population ageing in the future, with the speed of ageing in China being greater than that of India [32, 33]. The population ageing level of Japan is more acute than that of China and India, and Japan and China have greater speeds of ageing than India [33]. These previous conclusions are also in accordance with the conclusions in this study.

With the development of global economic and medical technology, the global spatial patterns of ageing have been re-formed. The differences in the ageing rates and total ageing populations in the 195 countries/regions expanded constantly from 1960 to 2017. In other words, the phenomenon of differentiation of global population ageing is becoming increasingly serious. The global heterogeneity in absolute terms of the total ageing population is larger than that in relative terms—the ageing rate—over the entire study period. This finding can be visualised in a spatiotemporal sequence map (Figs. 2 and 5) qualitatively, and it can be estimated quantificationally from the statistical results. In particular, the gap between the populous nations (e.g., China, India, the United States and Japan) and other countries is clearly widening in terms of the ageing rate and the size of the ageing populations.

Whether in absolute or relative terms, the quantile linear regression coefficient correlates positively with the quantile point. In other words, high ageing rate and ageing population quantiles have high linear growth rates, and vice versa. To some extent, the QRM results demonstrate the general rule of population change by age. Furthermore, the local trend of the ageing rate and ageing population in each country or region was estimated using BSTHM, which considered spatial structure (autocorrelation) and non-structure effects. This estimation revealed that although there were 44 countries and regions with low ageing levels or decreasing ageing rate trends, in absolute terms, the ageing populations in all 195 countries/regions showed an increasing trend. Specifically, the increase in the young population exceeded that of the old population in 44 countries and regions, but the old population continued to grow. In the other 151 countries and regions, the growth rates of the ageing populations exceeded those of the young populations. This phenomenon is more evident in developing or less-developed areas (e.g., Arica, Western Asia, South America). Fertility fell with unexpected speed in many less-developed countries, from an average of six children in 1950 to an average of two or three children in 2005 [5], while fertility in more-developed countries decreased from nearly three births per woman around 1950 to below two births per woman in the 1970s [5]. Most developed countries have had decades to adjust to their population ageing problem; however, many less-developed nations are experiencing a rapid increase of their aging populations within a single generation.

Population ageing will deeply affect health systems, e.g., public health policies and clinical management. A crucially increasing ageing population has led to new disease patterns. The prevalence of chronic diseases, including heart disease, arthritis, cancer and diabetes, has been rising in some ageing countries, e.g., Sweden, Japan, Denmark and the United States [34], since the 1980s [1, 35]. Therefore, the burden of chronic disease and late-life disease has also been increasing [2, 36]. Meanwhile, population ageing poses challenges to health care systems [37]. The challenges are seen already in many countries, e.g., China [37], the UK [38] and the United States [7]. Different countries and regions will face different challenges arising from population ageing. Particularly for poorer countries, more work is needed to understand the interrelationships between epidemiological and demographic change, as this is the basis of planning forthcoming health systems [39]. Our study provides a global perspective of spatiotemporal evolution, or trends, of population ageing. The results generated by this paper can help health policy makers assess the relative state of ageing among the 195 countries and regions worldwide. Furthermore, our findings can also provide references for developing international cooperative health measures to meet the challenges of global population ageing. Few researchers have studied the space-time trends of global ageing. We hope our statistical results serve as a basis of global disease mapping for the ageing population.

Although this paper explores the temporal and spatial evolution of population ageing globally from 1960 to 2017, it has some limitations. First, the law of historical evolution of the global population ageing is mainly investigated in our study. The prospective forecasting of spatiotemporal trends should be studied in the next work. Second, this paper does not consider the mechanisms of the space-time process of global population ageing and the influencing factors. This is something we will focus on in the future.

## Conclusion

QRM and BSTHM were employed to explore the spatiotemporal evolution of population ageing in 195 countries and regions. In addition, this paper considered the ageing population in absolute terms and the ageing rate in relative terms, leading to the following conclusions.

The proportion of ageing countries and regions with ageing (65 and over) percentages exceeding 7.0% increased from 19.0% (37/195) in 1960 to 46.7% (91/195) in 2017. Japan and Italy had the first and second highest ageing rate in 2017, 27.05 and 23.02%, respectively. At the continental level, Europe and Africa had the highest and lowest ageing levels from 1960 to 2017, respectively. Excluding Africa, the other five continents, Europe, Oceania, Asia, South America and North America, experienced fairly strong annual growth in their ageing rates (0.1532, 0.0873, 0.0834, 0.0723 and 0.0673%, respectively). The difference or heterogeneity of the ageing rate and ageing populations in the 195 areas has enlarged continuously; that is, the differentiation of global population ageing is becoming increasingly serious, especially in absolute terms.

Considering the overall spatial relative risk of population ageing, the 195 countries and regions can be classified into five ageing level grades, based on Jenks’ natural breaks classification. Fifty-five countries and regions received the top grade; except for Macedonia and Albania, all of the other 37 European countries, including those ranked in the top five in the world in ageing rate, received the highest grade. The other 18 areas with the highest grade are located in Asia (8), North America (6), South America (2) and Oceania (2). Most areas in Africa and some countries and regions in Asia and South America had low or lower ageing levels. There are 14 countries that exhibited both higher ageing rates and large ageing populations; this feature is most obvious in the United States. Four countries—Canada, Japan, Russia and Argentina—and nine countries in Europe have both high ageing levels and larger ageing populations. Eight countries have lower ageing levels but large ageing populations (most notably India).

Regarding local trends, 44 countries located mainly in Africa (26 countries) and Asia (15 countries) have both a declining trend in ageing rate and low ageing levels. Nevertheless, all 195 countries and regions showed increasing trends in their ageing populations. The other 71 countries and regions whose overall spatial relative risk of ageing rate was less than 0.95 showed positive local trends in their ageing rate. Nine European countries and the United States, all of which had the highest ageing levels, exhibited smaller average annual growth of their ageing rate. Additionally, an obvious spatial pattern of the local trend of ageing population could be seen, characterised by the north–south partition ‘low in north, high in south’. The absolute annual increase in ageing persons in China and India was larger than in all other areas, but the average annual increase rate was medium (2.74–3.28%), relative to the rest of the world.

## Abbreviations

BSTHM: Bayesian spatiotemporal hierarchical model
CAR: conditional autoregressive
CV: coefficient of variation
HPD: highest posterior density.
MCMC: Markov chain Monte Carlo
OLS: ordinary least squares
QRM: quantile regression model
